# Exploring﻿ electroencephalography with a model inspired by quantum mechanics

**DOI:** 10.1038/s41598-021-97960-7

**Published:** 2021-10-05

**Authors:** Nicholas J. M. Popiel, Colin Metrow, Geoffrey Laforge, Adrian M. Owen, Bobby Stojanoski, Andrea Soddu

**Affiliations:** 1 The Department of Physics and Astronomy, The University of Western Ontario, London, ON N6A 5B7 Canada; 2 Cavendish Laboratory, University of Cambridge, Cambridge, CB3 0HE UK; 3 The Brain and Mind Institute, The University of Western Ontario, London, ON N6A 5B7 Canada; 4 The Department of Psychology, The University of Western Ontario, London, ON N6A 5B7 Canada; 5 The Department of Physiology and Pharmacology, The University of Western Ontario, London, ON N6A 5B7 Canada; 6 Faculty of Social Science and Humanities, University of Ontario Institute of Technology, 2000 Simcoe Street North, Oshawa, ON L1H 7K4 Canada

**Keywords:** Computational science, Quantum mechanics

## Abstract

An outstanding issue in cognitive neuroscience concerns how the brain is organized across different conditions. For instance, during the resting-state condition, the brain can be clustered into reliable and reproducible networks (e.g., sensory, default, executive networks). Interestingly, the same networks emerge during active conditions in response to various tasks. If similar patterns of neural activity have been found across diverse conditions, and therefore, different underlying processes and experiences of the environment, is the brain organized by a fundamental organizational principle? To test this, we applied mathematical formalisms borrowed from quantum mechanisms to model electroencephalogram (EEG) data. We uncovered a tendency for EEG signals to be localized in anterior regions of the brain during “rest”, and more uniformly distributed while engaged in a task (i.e., watching a movie). Moreover, we found analogous values to the Heisenberg uncertainty principle, suggesting a common underlying architecture of human brain activity in resting and task conditions. This underlying architecture manifests itself in the novel constant K_Brain_, which is extracted from the brain state with the least uncertainty. We would like to state that we are using the mathematics of quantum mechanics, but not claiming that the brain behaves as a quantum object.

## Introduction

An important but outstanding issue in contemporary cognitive neuroscience is understanding the organizational properties of neural activity. For instance, is there a fundamental structure to the spatial–temporal patterns neural brain activity across different conditions? One common approach used to address this question is to examine the brain at “rest”. Measures such as functional connectivity, independent component analysis and graph theoretic metrics, have been applied to data recorded using different imaging techniques (e.g., functional magnetic resonance imaging (fMRI) and electroencephalography (EEG)), to cluster brain areas that exhibit similar activity patterns. Numerous studies have shown that brain activity during “rest” can be grouped into distinct networks across^1,2^; such as sensory (visual and auditory), default mode, executive, salience, and attentional (ventral and dorsal) networks that have been reliably reproduced across thousands of participants^3^, and are predictive of phenotypic measures like cognition and clinical diagnoses^4–6^. These results suggest these networks may be an intrinsic aspect of neural activity.

Indeed, the same set of structured patterns of neural activity have been found during "active" states, such as, while completing different tasks^7–9^. For instance, there is a high degree of correspondence between networks extracted during rest and those extracted during tasks measuring sensorimotor^10,11^ and higher-level cognitive abilities (i.e., working memory)^12,13^. Even completing a task as complicated as following the plot of a movie elicits the same network architecture as observed in the resting brain^14^. The correspondence between task and rest-based networks is so strong that task-based fMRI network activity can be predicted from the resting state^15^, and rest-task network pairs can be identified at the individual level^16^. Together, these results suggest that rest and task-based patterns of brain activity likely share a similar underlying neural architecture, despite distinct experiences and cognitive processes^17^.

There are, however, important differences between the patterns of brain activity elicited during rest and task-based paradigms, and the set of experiences and cognitive processes associated with each^18^. For instance, the presence or absence of a task is accompanied by increases in variability across different scales including neuronal firing rates changes in field potentials^19,20^, variation in fMRI blood oxygen level dependent (BOLD signal)^21^, and in EEG frequency bands^22^. Furthermore, through transcranial direct current stimulation (tDCS) it has been shown that frontal-lobe stimulation increases one’s proclivity to mind wander^23,24^. Importantly, these differences are associated with changes in properties of neural activity but not in changes in the underlying neural architecture.

Is there a way to identify the shared neural architecture underlying the cognitive processes associated with rest and active states while also quantifying how these processes diverge from that shared architecture of neural activity? In this paper, we applied mathematical methods analogous to those of quantum mechanics, and the concept of phase space to EEG recorded during rest and movie-watching to extract spatial and transitional properties of dynamic neural activity. Quantum mechanics was developed to describe the dynamics of the subatomic world in terms of probability amplitudes and densities of states. Quantum systems (in the Schrodinger formulation of quantum mechanics) are described by wavefunctions which square to a probability distribution leading to the loss of local determinism and the Heisenberg uncertainty principle (for an overview/intro to the subject see^25^). This uncertainty principle places a fundamental limit on the location and the momentum of a point particle^26^. In essence, if the position of a particle is known there is an underlying uncertainty in its momentum (one cannot precisely say how fast it is going) and vice versa. In addition to the adaptation of the wavefunction approach to quantum mechanics in this paper, we also employed a phase space model. Phase space is a widely used tool in the study of dynamical systems, where the positional variables are paired with their conjugate momenta which establishes a multidimensional space that describes all possible configurations of the given system. This space spans the entire range of states that a system can exist in, each point (in this hyper-space) represents a single state of the system. Phase space and its assorted formalisms are a classical concept, and we simply use it as another tool for analysing the EEG data. Herein, the mathematical methods of quantum mechanics are applied to EEG data to extract a proxy to phase space. This quasi-quantum approach naturally generates the concepts of ‘average’ position, ‘average’ momentum and culminates in an analogous Heisenberg uncertainty principle.

In this paper, we posit that using mathematical tools drawn from quantum mechanics, an underlying pattern representative of task and resting brain activity can be realised, in which differences across conditions are apparent, but culminates in a task independent constant value. It is important to note that we are not claiming that the brain behaves as a quantum object as some believe^27–30^. Rather, we have employed some of the analytical tools from the Schrodinger formulation of quantum mechanics to the brain with the aim of gaining new insight into resting and task-based brain dynamics. Not only does devising this model probe questions into the functions of the brain, but it also provides a novel approach to analysing the myriad of data available in neuroscience.

## Results

In this paper, we adapted the probability amplitudes of quantum mechanics to define new metrics for examining EEG data—the ‘average position’ and ‘average momentum’ of the EEG signal. These were constructed from our definition of ‘brain states’ based on the quasi-quantum model. This allowed us to ascertain the frequency with which unique brain regions are entered by the pseudo-wavefunction, as well as explore the average-valued phase space. Finally, an analogous uncertainty relationship to that of quantum mechanics was established, with the full mathematical derivation described in the methods.

### Average values

The ‘average position’ of the EEG data was first extracted performing a Hilbert transform of the pre-processed time courses, and then applying a normalization constraint. Typically, the Hilbert transformed data is used to generate a metric of power dispersion or to extract the phase of the signal^31–33^. Instead, we imposed a new normalization condition, thereby creating an analogy to the wavefunctions of quantum mechanics. Denoting the Hilbert transformed time course of the *j*th electrode as Ψ_j,_ this is equivalent to

1 $$\Psi_{j} \left( t \right) = A_{j} \left( {\text{t}} \right)\exp \left( {i \uptheta _{{\text{j}}} \left( t \right)} \right)$$

With $i = \sqrt { - 1}$. We then imposed the normalization condition,

2 $$\hat{\Psi }_{j} \left( t \right) = \frac{{\Psi_{j} \left( t \right)}}{{\sqrt {\mathop \sum \nolimits_{j = 1}^{92} \left| {\Psi_{{\text{j}}} } \right|^{2} } }}$$

The summation extends to 92, corresponding to the 92 electrodes selected from the original 129 on the head cap (channels removed from the face and neck for this analysis). This normalization constraint allowed us to define the probability at time *t* of the *j*th electrode as

3 $$P_{j} \left( t \right) = \hat{\Psi }_{j}^{*} \left( t \right) \times \hat{\Psi }_{j} \left( t \right)$$

With the * denoting complex conjugation^25^. We then can describe each moment in time as a ‘brain state’ that is fully described (in the context of this model) through the ‘wavefunction’. This ‘brain state’ uniquely specifies the EEG signal, and hence the dynamics of interest, at each moment in time. Using this definition of probability, we defined two average quantities of interest. The average position and momentum are given explicitly by,

4 $$\begin{aligned} \left\langle {x\left( t \right)} \right\rangle & = \sum\limits_{j = 1}^{92} {x_{j} P_{j} \left( t \right)} \\ \left\langle {p_{x} \left( t \right)} \right\rangle & = m\frac{d}{dt}\left\langle {x\left( t \right)} \right\rangle = {\text{m}} \sum\limits_{j = 1}^{92} {x_{j} \frac{d}{dt}P_{j} \left( t \right)} \\ \end{aligned}$$

With the same holding true for y. These two equations are how we create our quasi-quantum mechanical analogues. The second equation is an extension of Ehrenfest’s theorem, relating the average momenta of a particle to the time derivative of its average position. Where we have assumed a Hamiltonian with only a spatially dependent potential. Note that as the positions are fixed in space (positions of the electrodes) only the probability changes in time. Throughout this paper the mass *m* has been taking to be unity for both the *x* and *y* momenta. Each of the 92 electrodes were projected onto the horizontal plane, thus the *j*th electrode was described by one unique (*x*_*j*_, *y*_*j*_) point.

**Figure 1:**
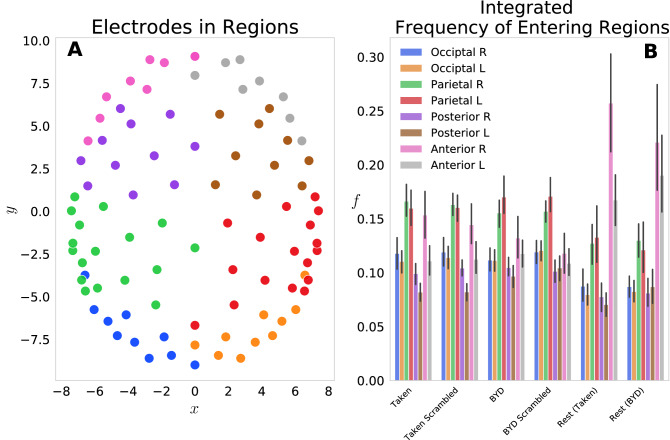
(**A**) Electrode locations for each of the 92 electrodes on the Electrical Geodesics Inc. headcap. Electrodes were projected onto a horizontal plane with the nose in the positive y direction. Electrodes have been colour-coded to display the constituent parts of the 8 groups for the frequency analysis, namely, occipital left (blue)/right (orange), parietal left (green)/right (red), posterior left (purple)/right (brown) and anterior left (pink)/right (grey). (**B**) Histograms representing the frequency of entering each region *f*_*G*_ are displayed for the six conditions tested. Significant within stimulus change is present between each of the Anterior Left and Right regions when comparing the pre-stimulus rest and the respective stimulated condition (*P* < 0.001, Tukey adjusted.). Error bars display the 1 standard deviation confidence interval.

We first examined this model by grouping the 92 electrodes into eight regions on the scalp: Anterior L/R, Posterior L/R, Parietal L/R, Occipital L/R and the probabilities of each electrode in the region were summed to give a region-level probability. Figure 1A shows the (*x*_*j*_, *y*_*j*_) locations of each electrode, with different colours representing each of the eight groups. Figure 1B displays the frequency of entering each region, grouped by the four task conditions and two resting conditions. This reflects the normalized count of regional probabilities integrated in time. We found that each anterior region was entered more frequently while at rest than when subjects were engaged in either movie. Specifically, the anterior left and right regions had significant within stimulus change, with *P* < 0.001 (Tukey adjusted) for the *Taken Rest*—*Taken*, *Taken Rest*—*Taken Scrambled*, *BYD Rest*—*BYD* and *BYD Rest*—*BYD Scrambled*. This is in line with Axelrod and colleagues’ findings which showed activation in the frontal region was associated with mind wandering^23,24^. We found frequency suppression in posterior regions, and an increase in anterior frequency in rest compared to the stimulated conditions, consistent with fMRI studies showing increased activation in the posterior cingulate cortex, and the medial prefrontal cortex during rest^22,24,34–37^. Thus, suggesting our model captures the frontal tendency associated with the brain activity while at rest.

### Phase space

**Figure 2:**
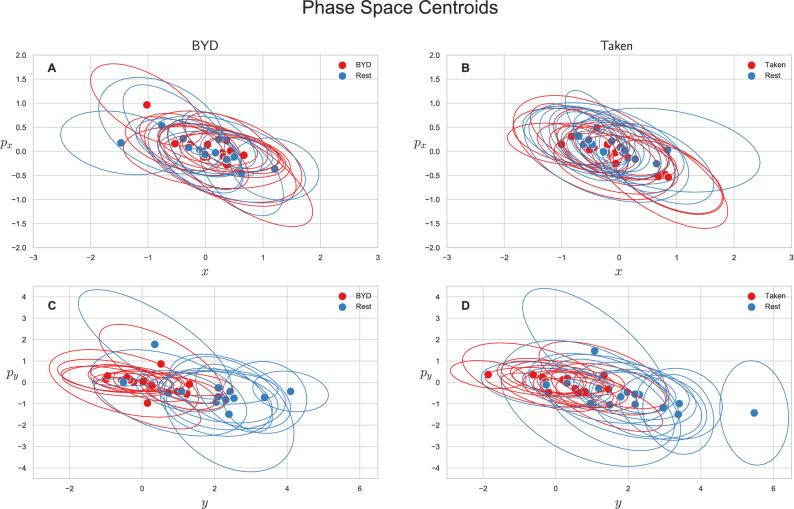
Mean phase space centroids for each subject. Ellipses represent the 1 standard deviation confidence interval. Centroids for the scrambled stimuli were omitted as they are indistinguishable from intact stimuli (*P* > 0.85) (**A**) Centroids for *“Bang! You’re Dead”* along the x direction. (**B**) Centroids for *“Taken”* along the x direction. (**C**) Centroids for *“Bang! You’re Dead”* along the y direction. (**D**) Centroids for *“Taken”* along the y direction. Differences are only apparent in the y direction (*P* < 0.001, Tukey adjusted) indicative of the higher level of anterior activation as noted in Fig. 1.

We also explored the average-valued phase space of this system. The phase space for each subject was plotted as the average position and momentum along the x direction $\left( {\left\langle {x\left( t \right)} \right\rangle ,\left\langle {p_{x} \left( t \right)} \right\rangle } \right)$ or as the average position and momentum along the y direction $\left( {\left\langle {y\left( t \right)} \right\rangle ,\left\langle {p_{y} \left( t \right)} \right\rangle } \right)$. Figure 2 shows the centroids of the phase space scatter plots for each subject with an ellipse representing the one standard deviation confidence interval. Note that values are only reported for the intact stimuli as an analysis of variance shows the scrambled and intact movies are indistinguishable in phase space (*P* > 0.85, Tukey adjusted). Figure 2A and B show the projection of the phase space centroid onto the plane spanned by $x$ and $p_{x}$ for *“Bang! You’re Dead”* and *“Taken”* respectively, and Fig. 2C and D ($y$,$p_{y}$) plane. The average position along the *y* axis $\left( {\left\langle y \right\rangle } \right)$ for the intact stimulus (*“BYD”* and *“Taken”*) and their scrambled forms are significantly different from the pre-stimulus rest counterparts with *P* < 0.001 (Tukey adjusted) whereas the task-positive and resting centroids are indistinguishable in the x plane (*P* > 0.05, Tukey adjusted). The averages of the group are reported in Table 1 along with their standard deviations. These values are the averaged value of the centroids (average of the within stimuli centre points in Fig. 2) for the respective position/momenta within each stimulus level. As also seen in Fig. 2C and D, there is a striking difference of one order of magnitude for $\left\langle y \right\rangle$ between the resting and task conditions, yet no marked differences in $\left\langle x \right\rangle$, $\left\langle {p_{x} } \right\rangle$, or $\left\langle {p_{y} } \right\rangle$.

**Table 1:** Group averages of the centroids.

Stimulus	$\left\langle x \right\rangle$	$\left\langle y \right\rangle$	$\left\langle {p_{x} } \right\rangle$	$\left\langle {p_{y} } \right\rangle$
Taken	$\left( { - 1.4 \pm 5.8 } \right) \times 10^{ - 1}$	$\left( {2.4 \pm 8.0} \right) \times 10^{ - 1}$	$\left( { - 5.8 \pm 27.0 } \right) \times 10^{ - 2}$	$\left( { - 1.0 \pm 4.1 } \right) \times 10^{ - 1}$
Taken Scrambled	$\left( { - 7.7 \pm 35.0 } \right) \times 10^{ - 2}$	$\left( {1.1 \pm 9.3 } \right) \times 10^{ - 1}$	$\left( {4.1 \pm 13.0 } \right) \times 10^{ - 2}$	$\left( {6.3 \pm 35.0 } \right) \times 10^{ - 2}$
Bang! You’re Dead	$\left( {1.2 \pm 4.7 } \right) \times 10^{ - 1}$	$\left( {3.5 \pm 74.0 } \right) \times 10^{ - 2}$	$\left( {2.6 \pm 33.0 } \right) \times 10^{ - 2}$	$\left( { - 3.0 \pm 42.0 } \right) \times 10^{ - 1}$
Bang! You’re Dead Scrambled	$\left( {1.4 \pm 5.7 } \right) \times 10^{ - 1}$	$\left( { - 2.6 \pm 7.5 } \right) \times 10^{ - 1}$	$\left( { - 1.5 \pm 2.8 } \right) \times 10^{ - 1}$	$\left( { - 5.5 \pm 53.0 } \right) \times 10^{ - 2}$
Rest (Pre-Taken)	$\left( { - 1.3 \pm 4.6 } \right) \times 10^{ - 1}$	$\left( {2.0 \pm 1.4 } \right) \times 10^{0}$	$\left( {9.1 \pm 19.0 } \right) \times 10^{ - 2}$	$\left( { - 6.3 \pm 7.3 } \right) \times 10^{ - 1}$
Rest (Pre-BYD)	$\left( {1.1 \pm 66.0 } \right) \times 10^{ - 3}$	$\left( {1.9 \pm 1.2 } \right) \times 10^{0}$	$\left( {1.0 \pm 26.0 } \right) \times 10^{ - 2}$	$\left( { - 4.3 \pm 7.5 } \right) \times 10^{ - 1}$

Significant differences are only noted for the rest acquired before Taken and Bang! You’re Dead when comparing the average y location to either of their task counterparts (scrambled and intact stimulus).

This analysis revealed two notable findings. First, there was a lack of significant differences in the momenta of the brain along the *x* and *y* direction. Second, the averages in momenta were not significantly different from 0 at the group level. The positive or negative momenta come from the competing time derivative of the probability and location of the electrode. Since the momenta average to 0, there is an equal number of anterior and posterior electrodes with both increases and decreases in probability.

Further, we examined changes in the probability values in both resting and active states. Animations of the probability distributions are present in Supplementary Material 1. In these animations, the differences in rest and task are apparent through the evolution of probability in time.

### Uncertainty principle

Despite the confirmation of previous neuroscientific results, and the apparent success of our quasi-quantum model, our research question as posed above remains only half answered. Using this model, we noted differences in the probability distributions and the phase space centroids in rest when compared to task. However, we still sought a parameter from the model that would remain the same in rest and task. To this end, we defined an analogous Heisenberg uncertainty principle of the form,

5 $$\Delta x\left( t \right)\Delta p_{x} \left( t \right) \ge K_{{{\text{Brain}}}}$$

**Figure 3:**
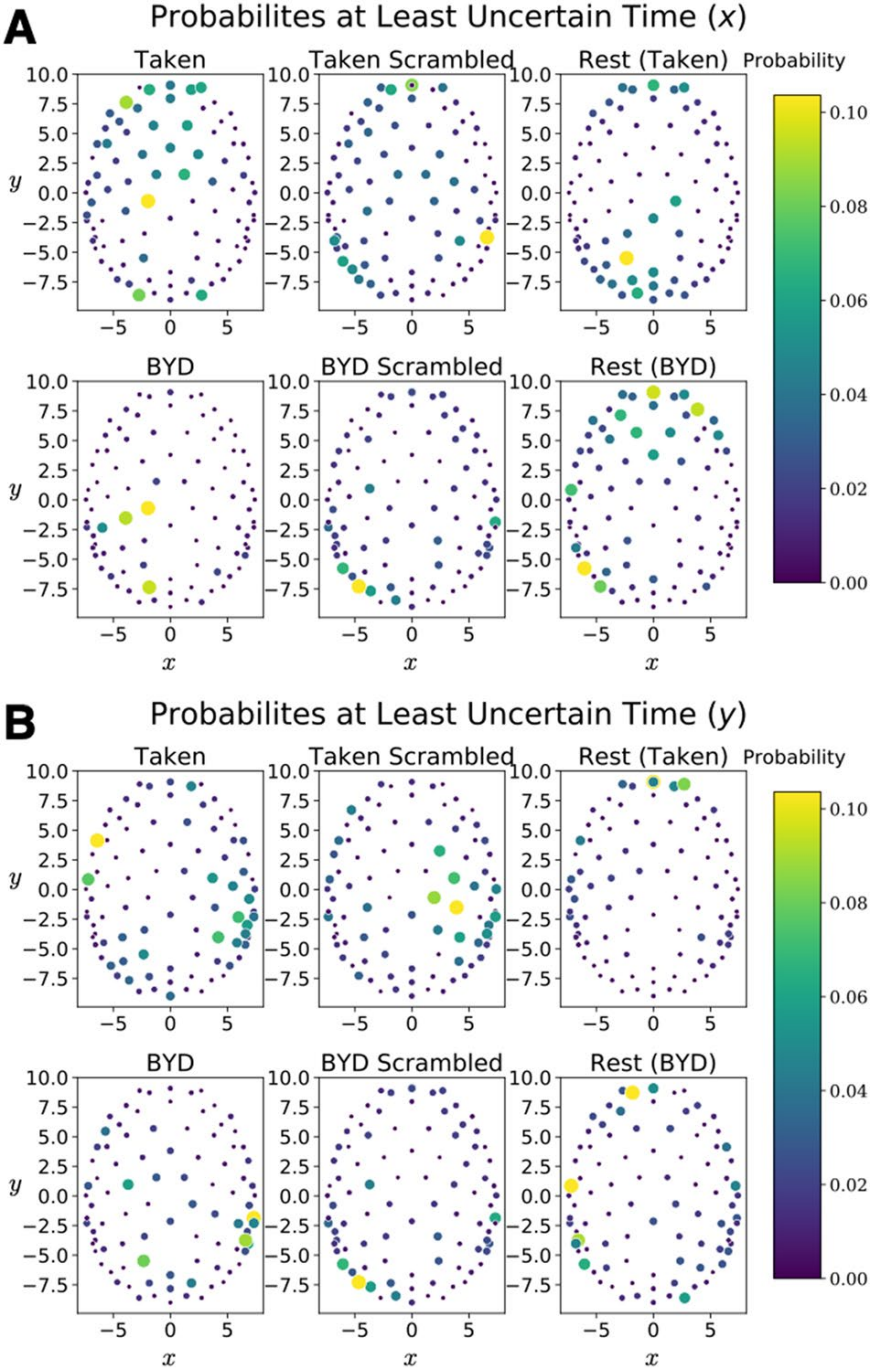
Probability maps corresponding to the least uncertain time point for each of the six experimental conditions. (**A**) The probabilities which lead to the minimum uncertainty as defined by the minimum of $\Delta x\left( t \right)\Delta p_{x} \left( t \right)$. (**B**) The probabilities which lead to the minimum uncertainty as defined by the minimum of $\Delta y\left( t \right)\Delta p_{y} \left( t \right)$. One subject is displayed for all Taken stimuli, and another for all Bang! You’re Dead stimuli.

Table 2 displays the values of this constant ($K_{{{\text{Brain}}}}$) acquired in all conditions, as well as the maximum value, mean value, and standard deviation. We found that this quasi-quantum model leads to a constant minimum value across $\Delta x\left( t \right)\Delta p_{x} \left( t \right)$ and $\Delta y\left( t \right)\Delta p_{y} \left( t \right)$ of $0.78 \pm 0.41 \frac{{{\text{cm}}^{2} }}{{4\;{\text{ms}}}}$ with (T = 0, *P* = 1). Note the unit of $\frac{{{\text{cm}}^{2} }}{{4\;{\text{ms}}}}$ is a result of the EEG being sampled at 250 Hz and the mass being taken to be unity. Furthermore, the average value and standard deviation of these quantities remains consistent across conditions with an average value of $9.3 \pm 4.4 \frac{{{\text{cm}}^{2} }}{{4\;{\text{ms}}}}$ (T = 0, *P* = 1) and a standard deviation of $18 \pm 29 \frac{{{\text{cm}}^{2} }}{{4\;{\text{ms}}}}$ (T = 0, *P* = 1). Notably, the maximum value does vary between conditions, with the largest value occurring while subjects watched the intact clip from *Bang! You’re Dead*. Despite the average position of the signal along the y direction being different in rest than during a task (*P* < 0.001), the quasi-quantum mathematical methodology leads to a constant uncertainty value. Quite remarkably, the values in the table display that the average uncertainty and minimum uncertainty is the same across different conditions, despite maxima varying by over two orders of magnitude. Thus, giving further credence to the idea that this uncertainty relation captures the similarities of the brain across the vastly different conditions. Figure 3 displays the probability distribution at the time corresponding to the minimum in uncertainty for both x and y.

**Table 2:** Various values extracted from the time courses of the products $\Delta x\left( t \right)\Delta p_{x} \left( t \right)$ and $\Delta y\left( t \right)\Delta p_{y} \left( t \right)$.

Stimulus	Minimum		Maximum		Average		Standard Deviation	
	$\Delta x \Delta p_{x}$	$\Delta y \Delta p_{y}$	$\Delta x \Delta p_{x}$	$\Delta y \Delta p_{y}$	$\Delta x \Delta p_{x}$	$\Delta y \Delta p_{y}$	$\Delta x \Delta p_{x}$	$\Delta y \Delta p_{y}$
Taken	$\left( {7.0 \pm 2.1 } \right) \times 10^{ - 1}$	$\left( {7.2 \pm 1.8 } \right) \times 10^{ - 1}$	$\left( {1.9 \pm 1.0 } \right) \times 10^{3}$	$\left( {1.4 \pm 0.8 } \right) \times 10^{3}$	$\left( {8.2 \pm 2.2 } \right) \times 10^{0}$	$\left( {8.2 \pm 2.2 } \right) \times 10^{0}$	$\left( {1.4 \pm 0.4 } \right) \times 10^{1}$	$\left( {1.3 \pm 0.4 } \right) \times 10^{1}$
Taken Scrambled	$\left( {6.4 \pm 2.6 } \right) \times 10^{ - 1}$	$\left( {6.8 \pm 2.1} \right) \times 10^{ - 1}$	$\left( {1.7 \pm 1.2 } \right) \times 10^{3}$	$\left( {2.1 \pm 2.2 } \right) \times 10^{3}$	$\left( {8.1 \pm 1.9 } \right) \times 10^{0}$	$\left( {7.8 \pm 2.0 } \right) \times 10^{0}$	$\left( {1.4 \pm 0.4 } \right) \times 10^{1}$	$\left( {1.4 \pm 0.7 } \right) \times 10^{1}$
Bang! You’re Dead	$\left( {7.6 \pm 4.9 } \right) \times 10^{ - 1}$	$\left( {7.5 \pm 3.1 } \right) \times 10^{ - 1}$	$\left( {0.1 \pm 3.1 } \right) \times 10^{5}$	$\left( {0.7 \pm 1.4 } \right) \times 10^{4}$	$\left( {9.4 \pm 6.7 } \right) \times 10^{0}$	$\left( {8.3 \pm 3.6 } \right) \times 10^{0}$	$\left( {4.1 \pm 8.9 } \right) \times 10^{1}$	$\left( {2.7 \pm 3.9 } \right) \times 10^{1}$
Bang! You’re Dead Scrambled	$\left( {7.4 \pm 3.2} \right) \times 10^{ - 1}$	$\left( {7.1 \pm 2.9 } \right) \times 10^{ - 1}$	$\left( {2.5 \pm 1.2 } \right) \times 10^{3}$	$\left( {2.5 \pm 1.6 } \right) \times 10^{3}$	$\left( {9.3 \pm 5.1 } \right) \times 10^{0}$	$\left( {8.6 \pm 4.4 } \right) \times 10^{0}$	$\left( {1.6 \pm 0.7 } \right) \times 10^{1}$	$\left( {1.5 \pm 0.8 } \right) \times 10^{1}$
Rest (Pre-Taken)	$\left( {9.7 \pm 4.2 } \right) \times 10^{ - 1}$	$\left( {1.1 \pm 0.6 } \right) \times 10^{0}$	$\left( {3.5 \pm 3.1} \right) \times 10^{2}$	$\left( {3.5 \pm 1.7} \right) \times 10^{2}$	$\left( {9.6 \pm 2.1 } \right) \times 10^{0}$	$\left( {1.3 \pm 0.4 } \right) \times 10^{1}$	$\left( {1.5 \pm 0.8 } \right) \times 10^{1}$	$\left( {1.9 \pm 0.7 } \right) \times 10^{1}$
Rest (Pre-BYD)	$\left( {6.3 \pm 3.7 } \right) \times 10^{ - 1}$	$\left( {8.6 \pm 6.1 } \right) \times 10^{ - 1}$	$\left( {3.7 \pm 2.0 } \right) \times 10^{2}$	$\left( {4.3 \pm 2.5 } \right) \times 10^{2}$	$\left( {8.7 \pm 3.3 } \right) \times 10^{0}$	$\left( {1.2 \pm 0.6 } \right) \times 10^{1}$	$\left( {1.4 \pm 0.5 } \right) \times 10^{1}$	$\left( {1.9 \pm 0.8 } \right) \times 10^{1}$

Considering the minimum value these products reach for each subject, we see a constant value in both x and y of $0.78 \pm 0.41\frac{{{\text{cm}}^{2} }}{{4\;{\text{ms}}}}$, an average value of the $9.3 \pm 4.4\frac{{{\text{cm}}^{2} }}{{4\;{\text{ms}}}}$, and a constant standard deviation of $18 \pm 29\frac{{{\text{cm}}^{2} }}{{4\;{\text{ms}}}}$.

The unit of $\frac{{{\text{cm}}^{2} }}{{4\;{\text{ms}}}}$ arises from the sampling of 250 Hz.

Maximum values differ across stimulation.

Values are reported as mean across subjects plus or minus 1 standard deviation.

## Discussion

In the current study, we investigated the spatial-extent and the associated transitional properties of neural activity in the brain during active and resting conditions, and whether similar underlying network properties exist. We found that applying the Hilbert transformation to the EEG data and normalizing it (Eq. 2) imposes a probabilistic structure to the EEG signal across the brain (Eq. 3), which we used to identify probability of spatial patterns of activity along with transitions in activity across the scalp. We found more anterior activity during rest relative to the movie watching, in both amplitude and phase space. This finding is in line with previous results showing increased activation in anterior region during rest^20–22,34,37,38^. Moreover, by normalizing the Hilbert transformed EEG signals and extracting average values akin to those of the wavefunction formulation of quantum mechanics, we were able to compute uncertainty in the ‘position’ and ‘momentum’ during rest and movie-watching, which is set by the new constant K_B_ = $0.78 \pm 0.41\frac{{{\text{cm}}^{2} }}{{4\;{\text{ms}}}}$.

It is alluring to associate the constant related to the ‘position’ and ‘momentum’ of neural activity to a fundamental principle, such as, the Heisenberg uncertainty principle. However, it is still unclear what this uncertainty means. It could imply limits to the degree to which the brain is accessible; increasing information about the precise location of the brain state (as described by our quasi-quantum ‘wavefunctions’) will produce a bigger uncertainty about where it will be at a subsequent time. These results offer an interesting perspective on the link between neural function and cognitive processes. For instance, as the ‘wavefunction’ becomes localized in space along a train of thoughts, we become distracted to increase the uncertainty, which may explain why minds wander and thoughts are fleeting?

Is the K_B_ value we found constant across different stimulus conditions, and independent of the number of electrodes used to acquire the data? To test this, we down sampled the EEG electrodes from 92 to 20 and performed the same analysis as in the main text. In line with 92 channels, we found the anterior tendency in rest, but we found reducing the electrodes to 20 resulted in a different constant K_B_ = $0.03 \pm 0.02\frac{{{\text{cm}}^{2} }}{{4\;{\text{ms}}}}$ (See Supplementary Material). This demonstrates that the model is able to capture the differences of rest/task, but a montage-dependent normalisation condition may need to be introduced.

It is important to note that uncertainty values of this form are inherent to any Fourier conjugate variables, as a value spreads out in one variable, it localizes in the other. This suggests that after defining the square of the Hilbert transformed EEG electrode time course to be the probability and imposing the properties of a Hilbert space onto the electrode signals, an uncertainty values can be extracted. In quantum mechanics, this uncertainty sets the limit for the scales that cannot be observed. This approach was inspired from the need in neuroscience for novel models to help interpret neuroimaging data. While this is an interesting methodological step forward, we still must determine if the observed uncertainty in the EEG data is supported by a new fundamental principle like in quantum mechanics, or if it is just the outcome of having built two new Fourier conjugate variables from the EEG signal.

Further work must be done to explore this constant with respect to the rich taxonomy of tasks and stimuli and varying states of consciousness that are routinely used in cognitive neuroscience. This methodology could be extended into fMRI, where the BOLD time courses could be Hilbert transformed creating a three-dimensional analogue of the EEG model presented in this paper.

Ultimately, this paper presented a novel methodology for analysing EEG data. Normalizing the data and treating it as a probability amplitude led to parameters that changed with the presence or lack of stimulus, while simultaneously establishing a constant value independent of stimulus. We have successfully applied a mathematical framework based on the formalisms of quantum mechanics to the resting and task paradigm in EEG (without claiming the brain is a quantum object). As neuroscience continues to evolve, the analytic tools at its disposal must also progress accordingly. We hope that this analytical tool, along with the advances in modelling and machine learning will aid in our understanding of the nature of consciousness.

## Methods

### Data acquisition

Twenty-eight healthy subjects were recruited from The Brain and Mind Institute at the University of Western Ontario, Canada to participate in this study. Informed written consent was acquired prior to testing from all participants. Ethics approval for this study was granted by the Health Sciences Research Ethics Board and the Non-Medical Research Ethics Board of The University of Western Ontario and all research was performed in accordance with the relevant guidelines/regulations and in accordance with the Declaration of Helsinki.

Two suspenseful movie clips were used as the naturalistic stimuli in this study. A video clip from the silent film “*Bang! You’re Dead*” and an audio excerpt from the movie “*Taken*” were shown to 13 and 15 subjects respectively in both their original intact and scrambled forms. Prior to the two acquisitions, a section of rest was acquired where the subjects were asked to relax, without any overt stimulation. Stimulus presentation was controlled with the Psychtoolbox plugin for MATLAB^39–41^ on a 15″ Apple MacBook Pro. Audio were presented binaurally at a comfortable listening volume through Etymotics ER-1 headphones.

EEG data were collected using a 129-channel cap (Electrical Geodesics Inc. [EGI], Oregon, USA). Electrode impedances were kept below 50 kΩ with signals sampled at 250 Hz and referenced to the central vertex (Cz). Using the EEGLAB MATLAB toolbox^42^, noisy channels were identified and removed, then interpolated back into the data. A Kolmogorov–Smirnov (KS) test on the data was used to identify regions that were not Gaussian. Independent components analysis (ICA) was then used to visually identify patterns of neural activity characteristic of eye and muscle movements which were subsequently removed from the data. EEG pre-processing was performed individually for each subject and condition.

Of the two movie clips tested, the first was an 8-min segment from Alfred Hitchcock’s TV silent movie “*Bang! You’re Dead*”. This scene portrays a 5-year-old boy who picks up his uncle’s revolver. The boy loads a bullet into the gun and plays with it as if it were a toy. The boy (and viewer) rarely knows whether the gun has a bullet in its chamber and suspense builds as the boy spins the chamber, points it at others, and pulls the trigger. As an alternative to visual stimulation, a 5-min audio excerpt from the movie “*Taken*” was also used. This clip portrays a phone conversation in which a father overhears his daughters’ kidnapping.

Furthermore, two “scrambled” control stimuli were used—one for each movie. This separates the neural responses elicited by the sensory properties of watching or listening to the movies from those involved in following the plot. The scrambled version of “*Bang! You’re Dead*” was generated by isolating 1 s segments and pseudorandomly shuffling the segments, thereby eliminating the temporal coherence of the narrative^14,43^. The scrambled version of “*Taken*” was created by spectrally rotating the audio, thus rendering the speech indecipherable^43,44^. The scrambled movie clips were presented before the intact versions to prevent potential carry-over effects of the narrative. Prior to subjects watching/listening to the scrambled stimulus a short segment of resting EEG was acquired.

### Model

Each of the *j* electrodes is described by an ordered pair (*x*_*j*_*, y*_*j*_*, z*_*j*_) in 3-dimensional space. To complete this analysis, the electrodes were first projected onto the (*x, y*) plane, removing the depth of the head. Figure 1A shows the locations of each electrode in this 2d-space. Following this projection, the time courses for each of the 92 electrodes were Hilbert transformed and then normalized following the procedure listed using Eq. (2). A probability was defined in this electrode-position space as the square of the Hilbert transformed time course (Eq. 3), analogous to the wavefunctions of quantum mechanics. Eight regions Anterior L/R, Posterior L/R, Parietal L/R, Occipital L/R) were then defined by grouping the 92 electrodes, and the frequencies of entering each region *f*_*G*_ were obtained by summing the probabilities electrodes within the group, then integrating in time.

6 $${\text{Prob}}_{G} \left( t \right) = \sum\limits_{k = 1}^{N} {\Psi_{k}^{*} \left( t \right) \times \Psi_{k} \left( t \right)}$$

$$f_{G} = \frac{1}{T}\sum\limits_{t = 1}^{T} {{\text{Prob}}_{G} \left( t \right)}$$

where each of the eight groups denoted by the subscript $G$ have a different number of constituent electrodes *N.* In the occipital left and right there are 10 electrodes each, in the parietal left and right there are 17 electrodes each, in the posterior left and right there are 10 and 11 electrodes respectively, and in the anterior left and right there are 8 and 9 electrodes respectively.

Upon getting the group level frequencies average values for position and momentum were calculated using Eqs. (4) and (5) (with identical expressions for y). Finally, to ascertain our analogous uncertainty principle, we sought expressions of the form

7 $$\begin{aligned} \Delta x & = \sqrt {\left\langle {x^{2} \left( t \right)} \right\rangle - \left\langle {x\left( t \right)} \right\rangle^{2} } \\ \Delta {\text{p}}_{x} & = \sqrt {\left\langle {p_{x}^{2} \left( t \right)} \right\rangle - \left\langle {p_{x} \left( t \right)} \right\rangle^{2} } \\ \end{aligned}$$

The expression for $\Delta x$ can be readily applied to the probabilities and positions as defined above, resulting in the first term given by

8 $$\left\langle {x^{2} \left( t \right)} \right\rangle = \sum\limits_{j = 1}^{92} {P_{j} \left( t \right) x_{j}^{2} }$$

And the second term given by the square of Eq. (4). The second term of $\Delta {\text{p}}_{x}$ is given by the square of Eq. (5), but the first term is more nuanced. This is owing to the complex number returned when acting the derivative operator twice on the probability. To overcome this, Fourier transforms were used to change Eq. (5) into the momentum basis which then allowed for the efficient calculation of $\left\langle {p_{x}^{2} \left( t \right)} \right\rangle$. Denoting $\tilde{\rm P}_{j} \left( t \right)$ as the momentum-space probability obtained through a 2-dimensional, non-uniform Fourier transform of the position space pseudo-wavefunction, Eq. (5) can be rewritten as,

9 $$\left\langle {p_{x} \left( t \right)} \right\rangle = \sum\limits_{j = 1}^{92} {\tilde{\rm P}_{j} \left( t \right)p_{j} }$$

Leading to the first term in the ${\Delta p}_{x}$ expression to be written as,

10 $$\left\langle {p_{x}^{2} \left( t \right)} \right\rangle = m^{2} \sum\limits_{j = 1}^{92} {\frac{{x_{j}^{2} }}{{\tilde{\rm P}_{j} \left( t \right)}}\left[ {\frac{d}{dt}P_{j} \left( t \right)} \right]^{2} }$$

**Figure 4:**
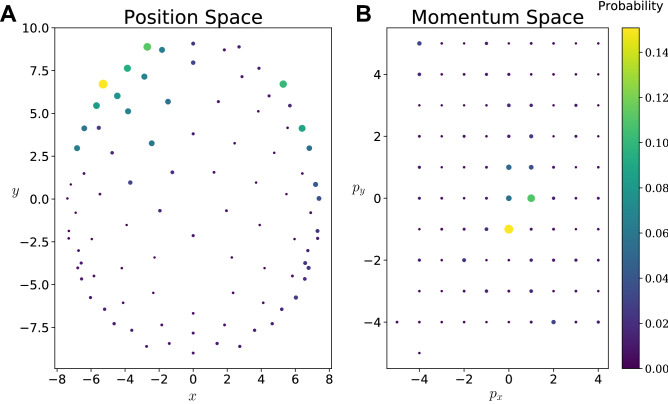
(**A**) Probability distribution for a single subject in the position basis. (**B**) Momentum basis probability distribution for a single subject. The momentum values used for the Fourier transform are indicated by the point locations. Points are colour-/size-coded to represent the probability value at that location.

The FINUFFT python wrapper was used to take the Fourier transform using a type 3, 2d non-uniform FFT^45,46^, and the minimum value in time of the uncertainty relation was found. Points in momentum space were sampled on $p_{x} \in \left[ { - 4,4} \right]$ and $p_{y} \in \left[ { - 4,5} \right]$ along with the two additional points (− 5, − 4) and (− 4, − 5). Figure 4 shows the position and momentum probabilities respectively in their own basis. An animation showing how these evolve in time for the different conditions is presented in Supplementary Material 2.

To compute the values reported in Table 2, the corresponding value was found for each subject, and these were used to calculate the group average reported here.

## Supplementary Information

Supplementary Figures.

Supplementary Information.
