# Supplementary material for: Exploring﻿ electroencephalography with a model inspired by quantum mechanics
Source: Sci Rep. 2021 Oct 5;11:19771. doi: 10.1038/s41598-021-97960-7 (PMC8492705; doi:10.1038/s41598-021-97960-7)
Supplement: Supplementary file 1 — Supplementary Figures. [file 41598_2021_97960_MOESM1_ESM.docx]

Supplementary Material 1

To explore how the probability evolves in time, and if there are visible differences in rest and task, we have made animations of the probability distribution. These animations illustrate the probability as defined by our model at each electrode. The probability is encoded in both the size and color of the electrode. Figure S1 is Taken, Figure S2 is Taken Scrambled, and Figure S3 is the rest acquisition prior to Taken.


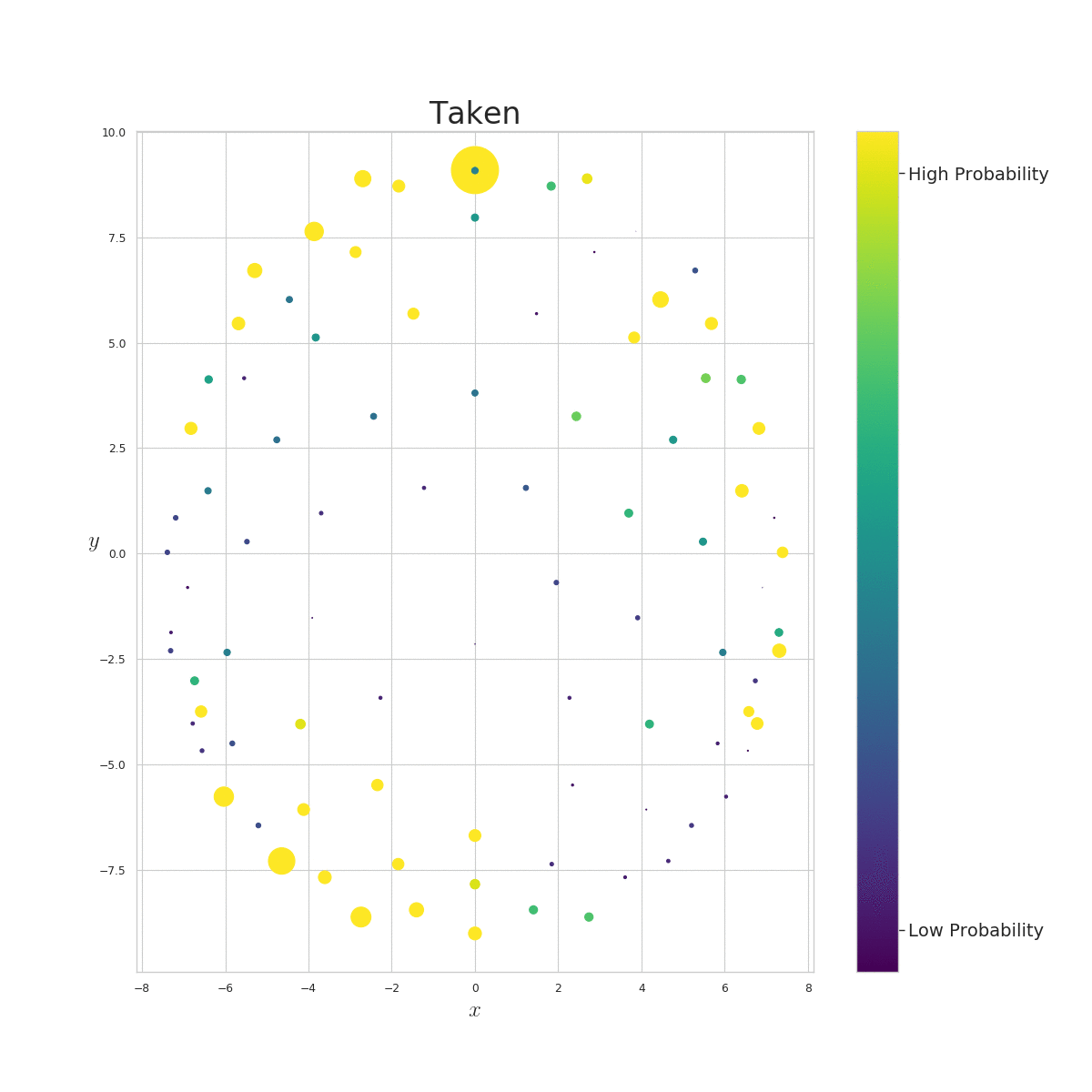


Figure S1 Animation of probability at each electrode for the Taken acquisition.


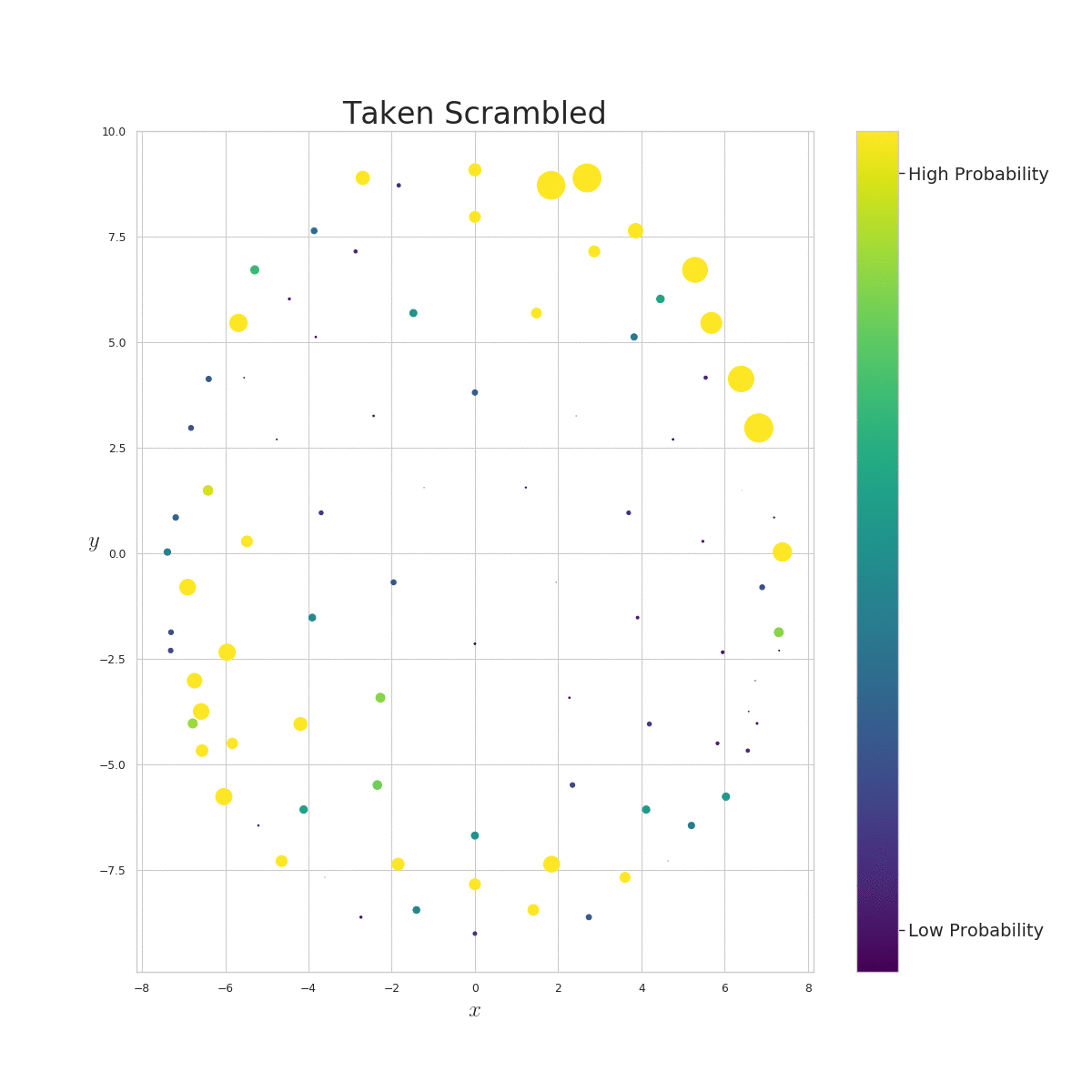


Figure S2 Animation of probability at each electrode for the Scrambled Taken acquisition.


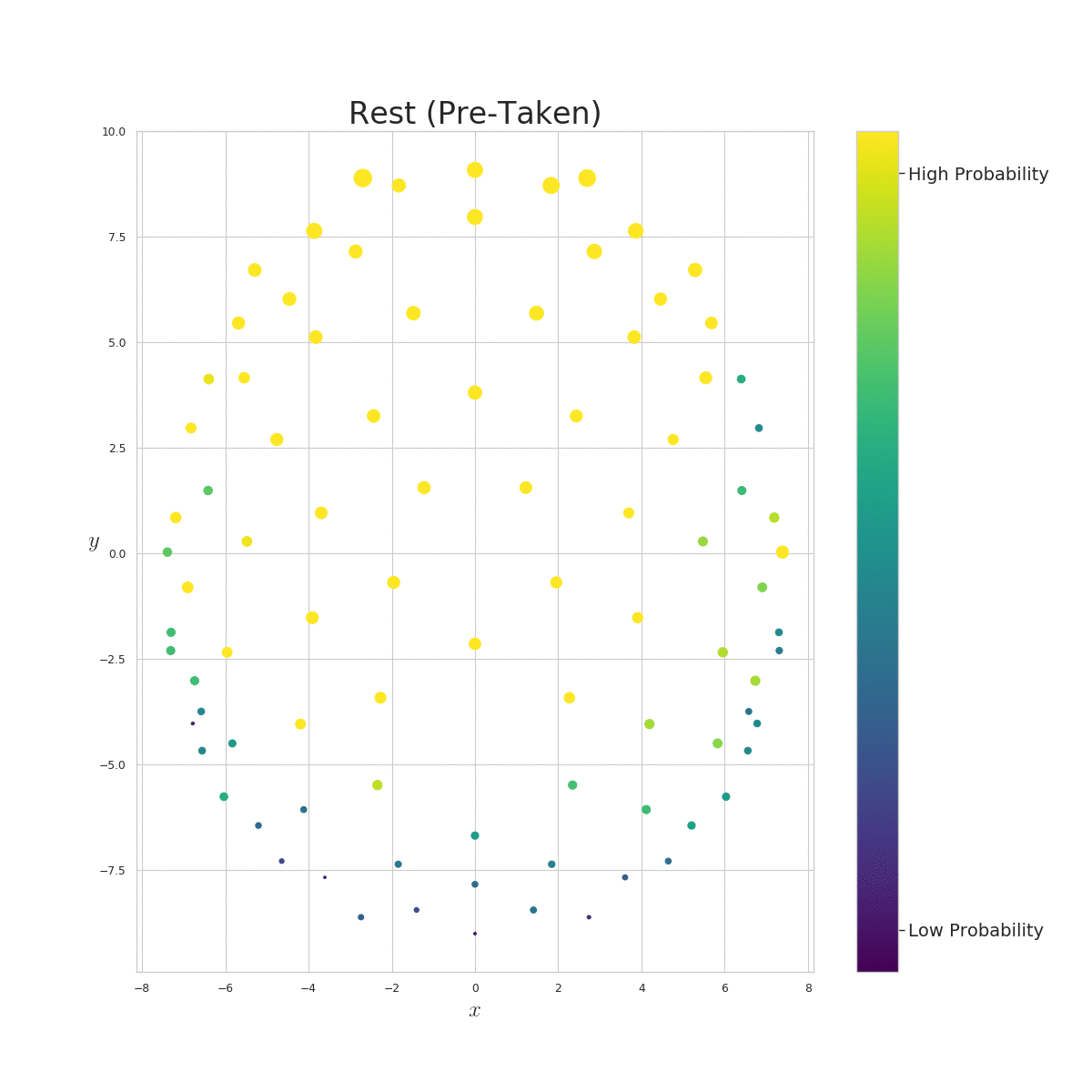


Figure S3 Animation of probability at each electrode for the rest prior to Taken.

Similarly, Figure S4 is Bang! You’re Dead, Figure S5 is the scrambled version of Bang! You’re Dead, and Figure S6 is the rest acquisition prior to Bang! You’re Dead.


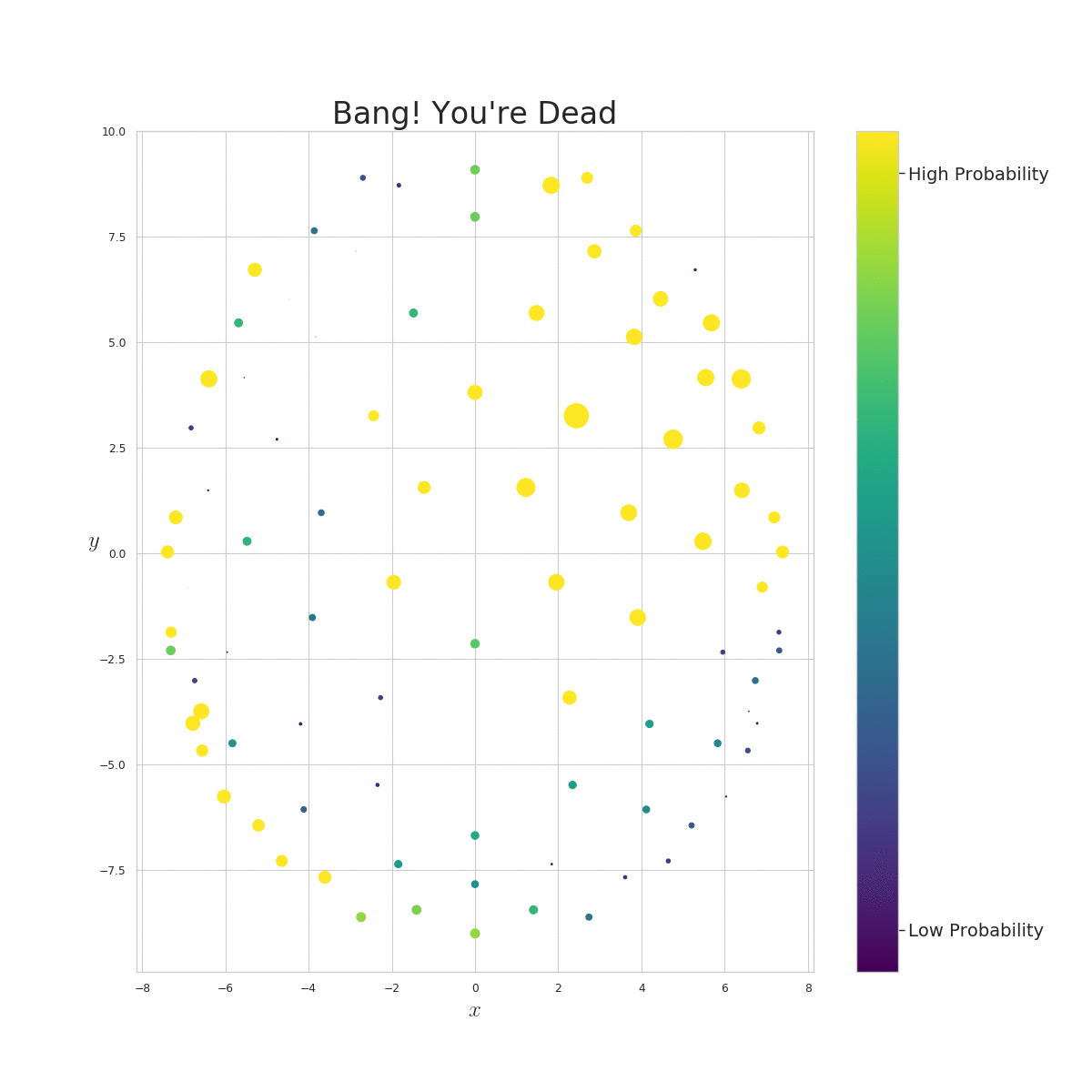


Figure S4 Animation of probability at each electrode for the Bang! You’re Dead acquisition.


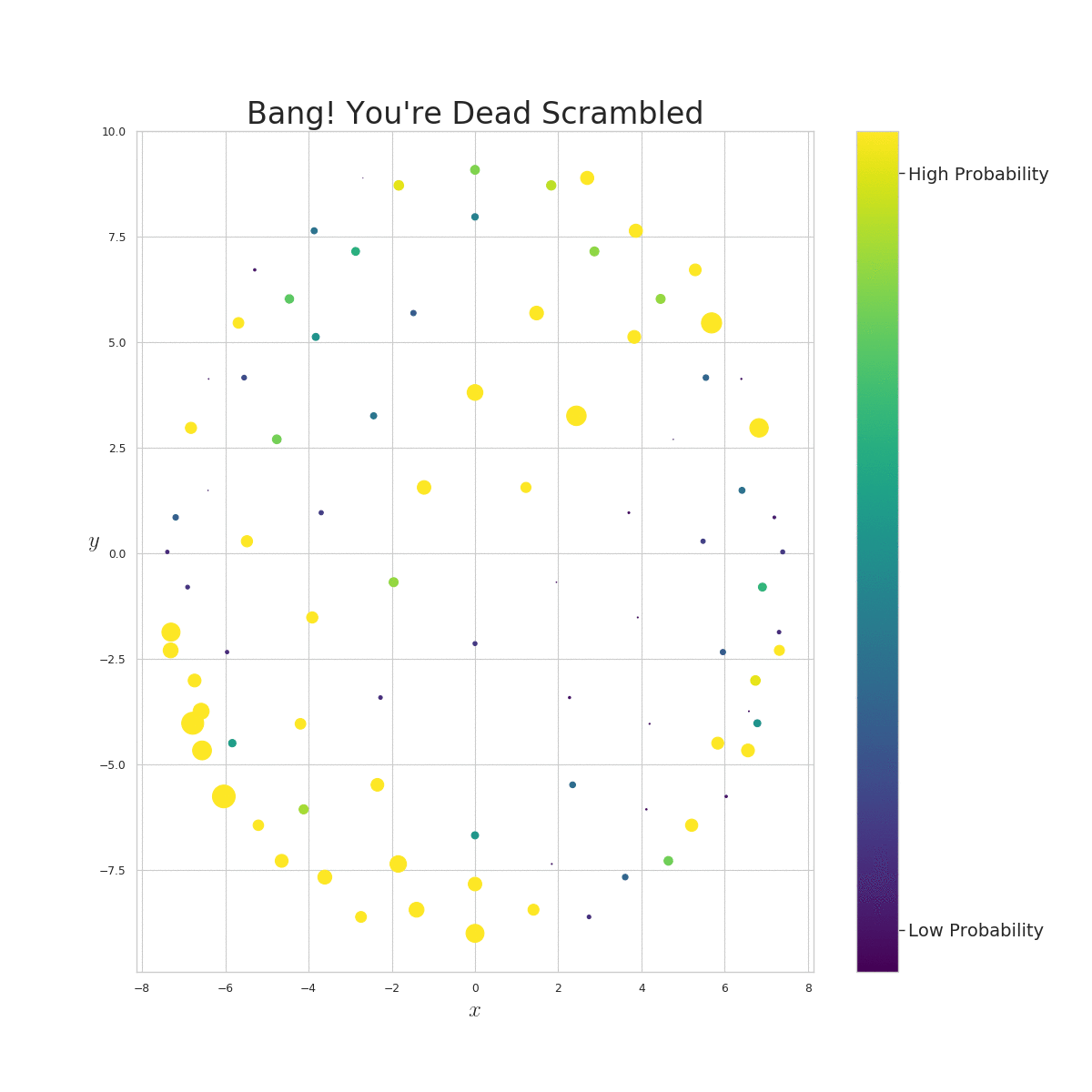


Figure S5 Animation of probability at each electrode for the Scrambled Bang! You’re Dead acquisition.


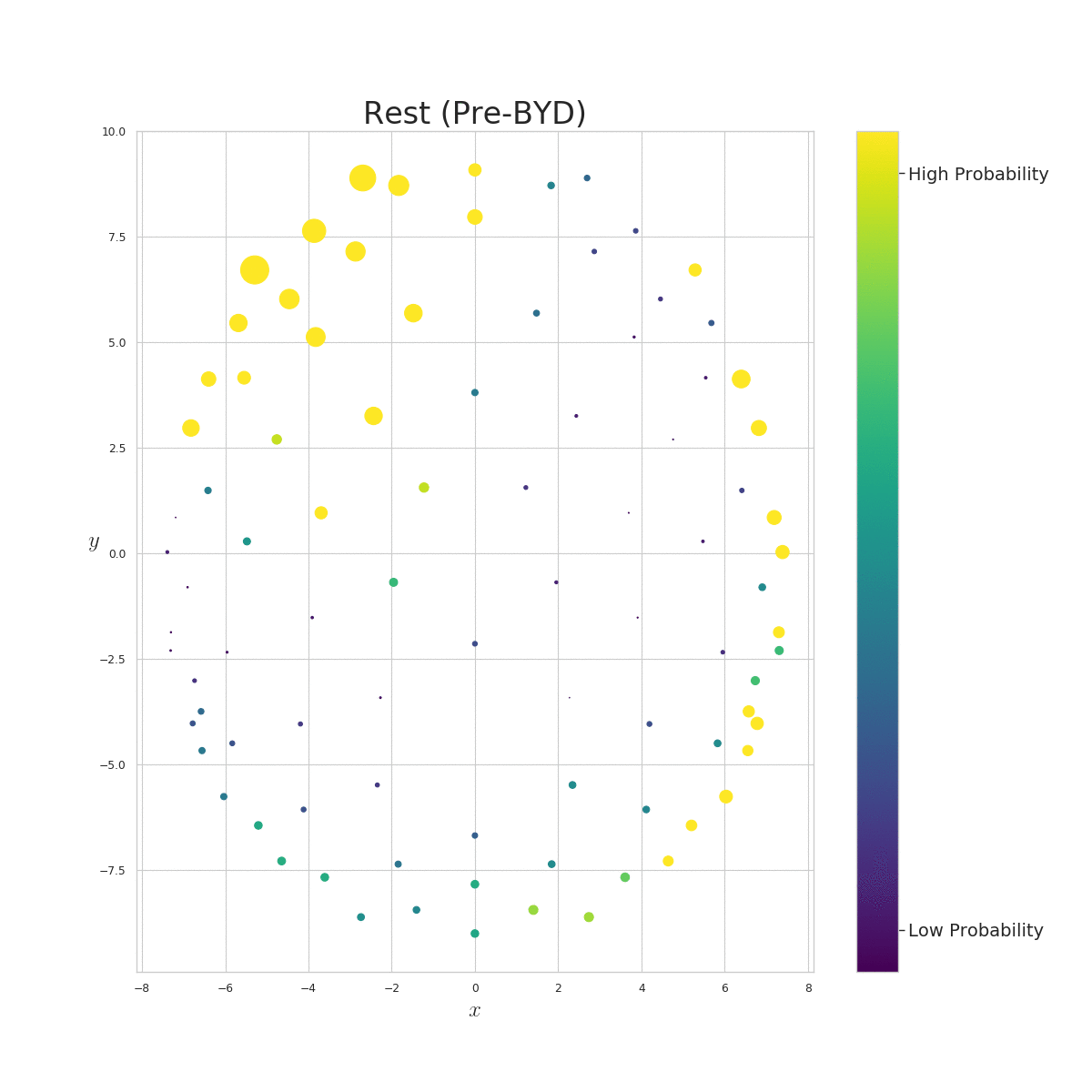


Figure S6 Animation of probability at each electrode for the rest prior to Bang! You’re Dead.
