# Supplementary material for: Exploring﻿ electroencephalography with a model inspired by quantum mechanics
Source: Sci Rep. 2021 Oct 5;11:19771. doi: 10.1038/s41598-021-97960-7 (PMC8492705; doi:10.1038/s41598-021-97960-7)
Supplement: Supplementary file 2 — Supplementary Information. [file 41598_2021_97960_MOESM2_ESM.docx]

Supplementary Material 2

To verify how our model worked with differing numbers of electrodes, we down sampled the 92 electrodes to a smaller subset of 20. Herein all of the same figures as the main text are presented for this smaller montage.

We were able to confirm the result of the anterior tendency in rest when compared to task. This is clear from Figure S7.
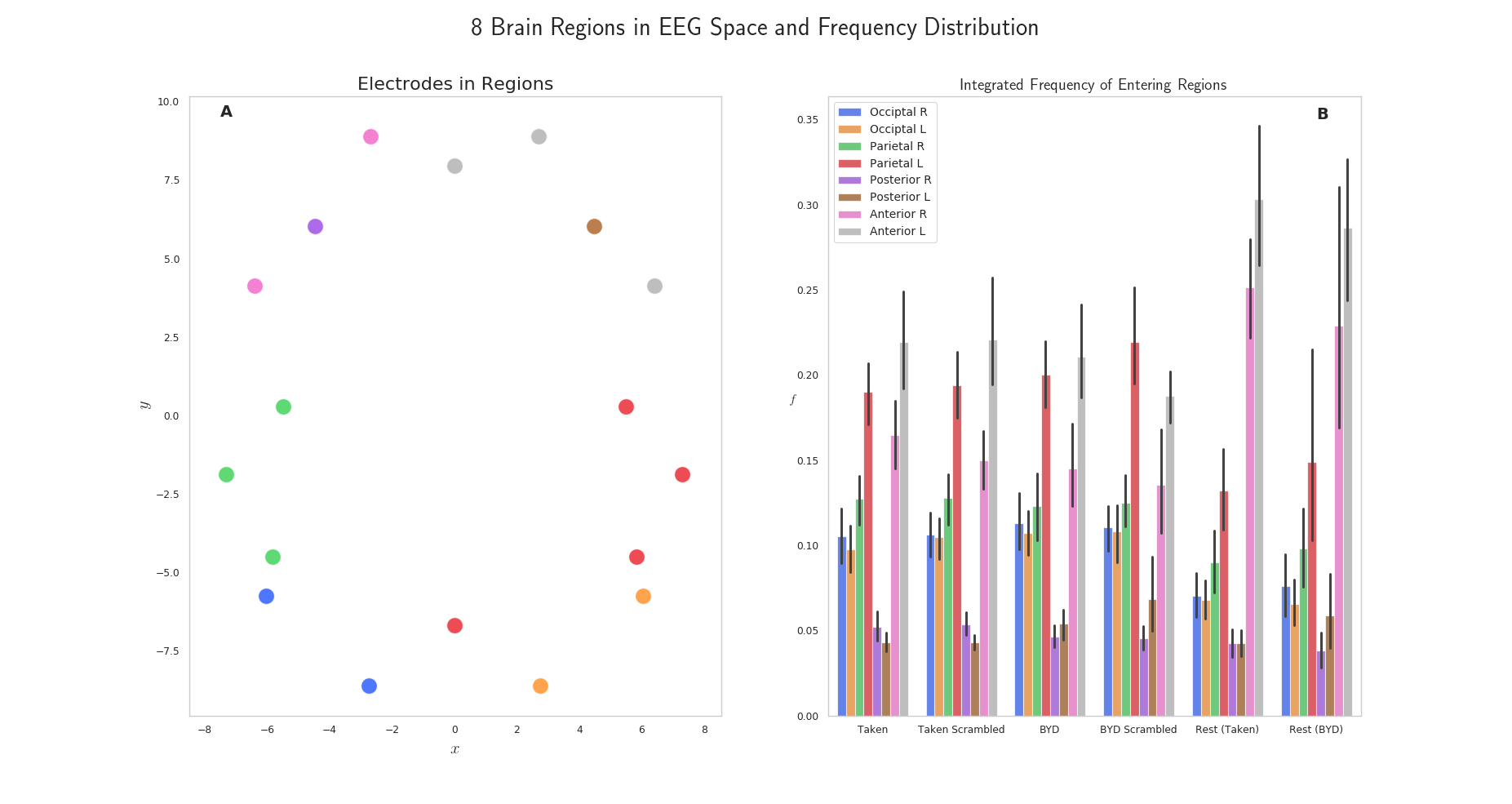


Figure S7 **A.** Electrode locations and their respective groups for the 20-electrode montage. **B.** Integrated probability distribution in time for the down-sampled 20 electrode montage. The anterior tendency of rest is noted again in this smaller montage.

Next, we turned our attention back to the average-valued phase space of this system. Again, the same tendency for an anterior increase in rest, and momenta averaging to 0 was observed as demonstrated in Figure S8.

Figure S8 Mean phase space centroids for each subject with the 20-electrode montage. Ellipses represent the 1 standard deviation confidence interval. **A:** Centroids for “Bang! You’re Dead” along the x direction. **B:** Centroids for “Taken” along the x direction. **C:** Centroids for “Bang! You’re Dead” along the y direction. **D:** Centroids for “Taken” along the y direction. Differences are only apparent in the y direction (P$<$0.01, Tukey adjusted) indicative of the higher level of anterior activation as noted in Figure 1.

Finally, we wanted to verify the presence of the constant. Results for this are summarized in Table S1, but a minimum uncertainty value of $0.03\pm0.01\frac{cm^{2}}{4ms}$ is seen across all conditions.

Table S1 A constant minimum uncertainty of $0.03\pm0.01\frac{cm^{2}}{4ms}$ is observed. Different than the value of $0.78 \pm0.41\frac{cm^{2}}{4ms}$ reported for the 92-electrode montage in the main text, but a constant value, nonetheless.


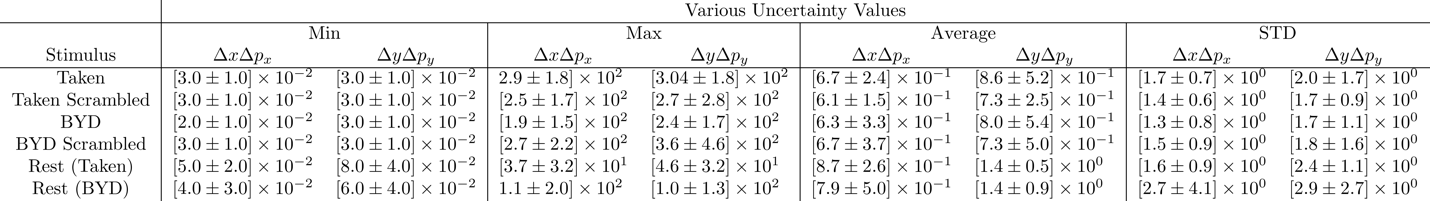


It is evident that we still have a constant value, although it is now smaller. If we believe that there is truly a constant value, somehow limiting the brain, then some form of normalization condition that is montage dependent is necessary. It is still unclear what the significance of the constant is, although it is clear that this model has an inherent uncertainty as defined in the main text.
